# Influence of Brain Gym Activities on Sleep Quality in Moderate Insomnia

**DOI:** 10.7759/cureus.28693

**Published:** 2022-09-02

**Authors:** Nikita H Seth, Pratik Phansopkar, Sakshi K Kariya

**Affiliations:** 1 Physiotherapy, Ravi Nair Physiotherapy College, Datta Meghe Institute of Medical Sciences, Wardha, IND; 2 Musculoskeletal Physiotherapy, Ravi Nair Physiotherapy College, Datta Meghe Institute of Medical Sciences, Wardha, IND

**Keywords:** pittsburgh sleep quality index, insomnia severity scale, insomnia, brain gym, sleep

## Abstract

Mental health has suffered considerably as a result of advancing time and technological developments. Poor quality of sleep affects people of all ages, and non-pharmacological remedies are becoming increasingly important. Nearly 60% of all undergraduate students are reported to have a poor quality of sleep, with 7.7% fitting the criteria for insomnia. Sleep deprivation is found to affect the immune function, brain maturation, development of the body, metabolic process, and cognition, as well as maintaining normal homeostasis of the body. Sleep quality and quantity have a severe influence on learning and memory and thus a major influence on students’ quality of life. Brain gym exercises are a formidable contender in this race. Still, further study is required before a solid conclusion can be formed on its value as an intervention, so to evaluate the effect of brain gym exercises as a non-pharmacological measure, this study was conducted with a total of 65 participants based on the inclusion and exclusion criteria with the duration of practice as five days in a week with a session of 25 minutes with adequate intervals. An insomnia rating scale (IRS) is used to get the desired population for giving the intervention, along with the Pittsburgh Sleep Quality Index (PSQI). This study was conducted at the Physiotherapy College of Wardha. The results were given by statistical analysis using descriptive and inferential statistics. The data analysis depicted that after the brain gym activity intervention, there was a marked reduction in the score of PSQI, suggesting significant improvement in their sleep quality, and it can be used as non-pharmacological management for mild to moderate insomnia students.

## Introduction

As a response to changing times and new advancements, mental health has suffered greatly. Sleep deprivation affects our mental health, motivation, reasoning, and understanding of events [1]. People of all ages are affected by poor sleep quality. Nearly 60% of all undergraduate students are reported to have poor quality sleep, with 7.7% fitting the criteria for insomnia. Sleep issues have a major influence on students’ daily life. Students experience different changes and have to cope with various stress and changing environments, and this costs them the loss of their peace of mind and disturbance in sleep patterns [2].

Sleep is defined as a functional state that comprises of behavioral and physiological processes. It has certain manifestations, such as relative immobility, cyclic pattern, and an increase in the threshold of response to external stimuli. The functions of sleep include immune boost, brain maturation, and development of the body, modulating metabolic processes at a molecular level, increasing performance, and maintaining catecholamine in the brain [3]. According to recent data, poor quality sleep and insufficient amount of sleep have been found to contribute as high risk factors for health outcomes in developing countries on the basis of the quality of life. Sleep deprivation is found to affect the immune function, brain maturation, development of the body, metabolic process, and cognition, as well as normal homeostasis of the body. Sleep quality and quantity have a severe influence on learning and memory [4]. According to experts, sleep enhances memory and thinking skills in two ways. For starters, a sleep-deprived person cannot study effectively. Furthermore, sleep itself plays a role in memory consolidation, which is necessary for acquiring new information. Sleep deprivation can be total, suggesting no sleep at all, partial, implying either early or late sleep is denied, or selective, implying that certain phases of sleep are deprived [5]. The sleep-wake cycle is governed by two separate biological processes in the body that interact and balance one another. This concept, which was proposed in the early 1980s by Swiss sleep researcher Alexander Borbely, is also known as the two-process model of the sleep-waking cycle. It encompasses the circadian rhythm as well as sleep-wake equilibrium. The circadian rhythm governs the body’s internal activities as well as its degree of awareness. Both of these processes are affected to some extent by food, drugs, exercise, and daily schedule [6].

Among these exercises in the brain gym are good contenders in the competition. However, there is still more to be done before a definitive conclusion can be made about its effectiveness as an intervention. Dennison and Dennison invented educational kinesiology which is another name for brain gym exercises [7]. It was developed in the 1970s, and it consists of a series of movements that stimulate the brain, encourage neurological reprogramming, and aid whole brain learning In order to operate normally, healthy individuals require between seven and a half to eight hours of sleep every night. There is various evidence on the effectiveness of brain gym and brain training intervention on working memory performance of students. It has been found that brain training and brain gym exercises were successful interventions in enhancing working memory performance with a significant increase in visual-spatial skills. 

## Materials and methods

After getting clearance from the Institutional Ethics Committee (IEC) the study was started. The study design was a quasi-experimental study with mild to moderate insomnia students as participants of the study. The study period was six months, from June 2021 to February 2022. Inclusion criteria were age group of 18 to 24, physiotherapy undergraduate students, and moderate severity insomnia (15-24 score on the Insomnia Severity Index). Exclusion criteria were students with migraines, students who were diagnosed with a psychological disorder and undertaking psychotic drugs, a history of neurosurgery, and cognitive damage.

The sample size was 65, so the total numbers of participants screened were 120 by using the Insomnia Severity Index, out of which 65 participants were selected based on the inclusion and exclusion criteria without specific gender distribution. The participants (n=65) were well informed about the research, and informed consent was taken. They were then given pre and post-interventional assessments using the Pittsburgh Sleep Quality Index (PSQI) scale, which was self-administered, with a 86.5% specificity and 89.6% sensitivity. Readings were recorded and exercise interventions were given for the month. The targeted population was physiotherapy students with moderate insomnia because of the feasibility. All the participants actively participated and were able to successfully complete the exercise program. Statistical analysis was done using descriptive and inferential statistics using Wilcoxon signed rank test, and Spearman’s rank order correlation, and the software used in the analysis was SPSS version 17.0 (IBM Inc., Armonk, New York). A p-value of <0.05 is considered significant.

The brain gym exercise protocol is outlined in Table 1. 

**Table 1:** Intervention design and effects of the brain gym exercises

	Brain gym exercises	Effects
1	Spot marching	Warm-up exercise
2	Hook ups	Helps in mind and body relaxation
3	Positive points	Helps to improve memory and reduce stress levels
4	The active arms	Helps in activating the brain for diaphragm relaxation; improves hand-eye coordination and tool-controlling skills
5	Earth buttons	Helps in improving mental alertness and whole body orientation
6	The energy yawn	Perfect exercise to improve oxygenation
7	Lazy eights	Helps in boosting eye muscle control, balance, and concentration
8	Gravity glider	Improves the blood and oxygen flow; boosts confidence and improves stability
9	Foot flex	Improves posture and socialization. It also helps in relaxation
10	The energizer	Helps in improving posture; keeps the back muscle toned and the spine supple, flexible, and relaxed

**Figure 1:**
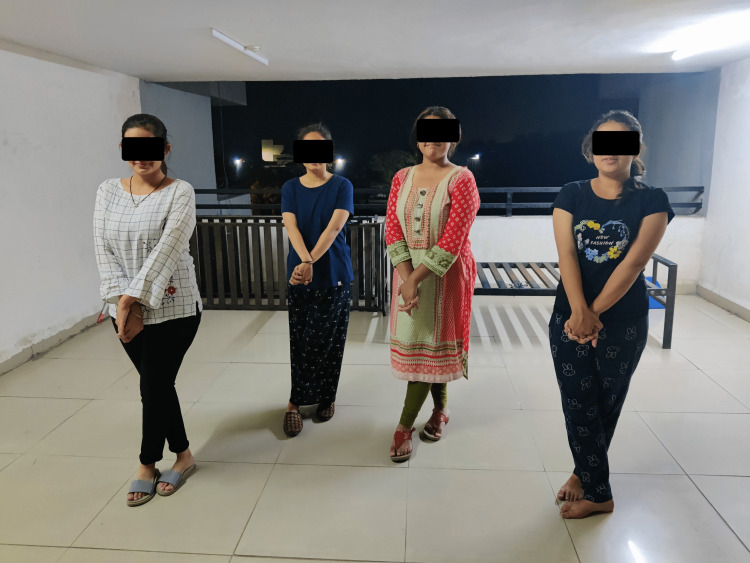
Participants demonstrating the hook ups exercise

**Figure 2:**
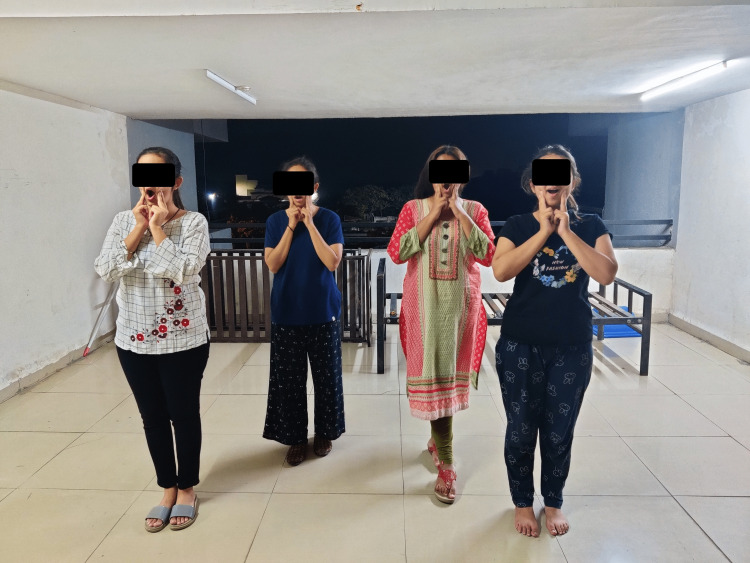
Participants performing the energy yawn exercise

Spot marching is done in the beginning as a warm-up, in which the subject stands straight and lifts both legs continuously above the ground for the time duration of one minute. The hook ups exercise is done by standing while crossing the hands and keeping them near the chest, and taking a deep breath with eyes closed and feet interlocked just like hands. The participant is advised to keep on breathing deeply. This is done for duration of two minutes (five sets of eight repetitions) (Figure 1). Positive points help to reduce stress levels. The subject is instructed to breathe deeply and gently press the eyeballs with eyes closed for duration of one minute (10 repetitions). It also stimulates lateral and side-to-side coordination. Earth buttons helps in improving mental alertness and whole body orientation. Earth buttons are situated on the body’s front midline, which serves as the central point of reference for all activities involving both sides of the body. Rest two fingers of one hand on the lower lips and place the palm of the other hand over the navel to do this. Breathe deeply while looking at the floor, and look at the ceiling and floor gradually just by moving the eyes. This is performed for two minutes (10 repetitions). The energy yawn is a perfect exercise to improve oxygenation. It can be performed in standing or sitting. Place the index and middle finger over the jaw muscles on both sides, rub the jaw muscles gently like massaging but with adequate pressure, then open your jaw in a long yawning motion then gently close the jaw. This is performed for one minute (10 repetitions) (Figure 2).

**Figure 3:**
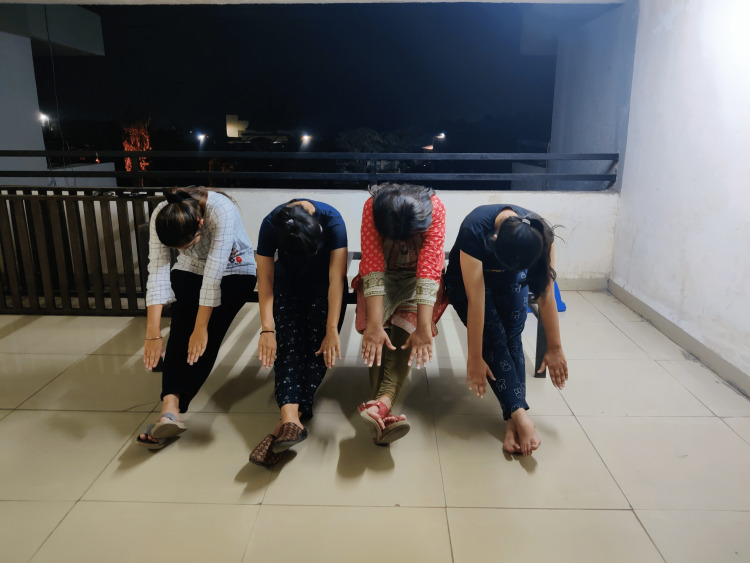
Participants performing gravity gliders exercise

**Figure 4:**
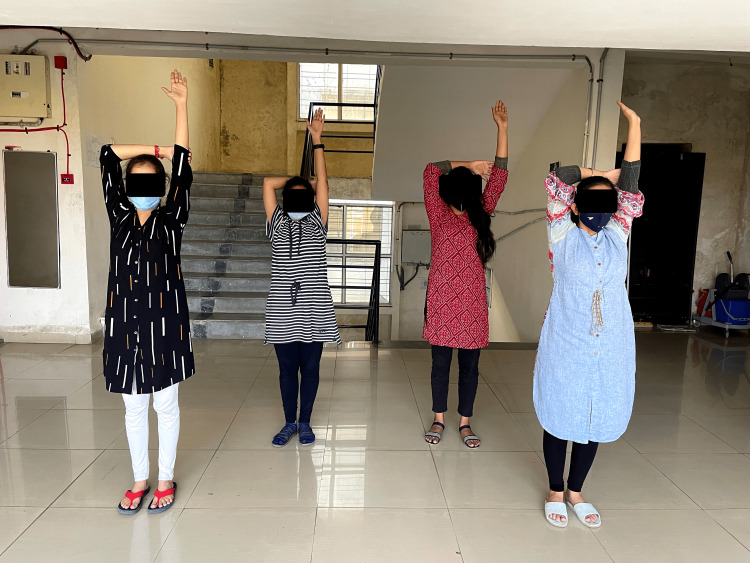
Participants performing arm activation exercise

Lazy eights help in boosting eye muscle control, balance, and concentration. The participant is instructed to extend the hand and make a figure of eight horizontally in front for a duration of one minute (10 repetitions). The gravity glider works by improving blood and oxygen flow. It boosts confidence and improves stability. The participant is instructed to sit comfortably by crossing the ankles and keeping the knees relaxed, bend forward to reach out in front, and letting the arms glide down while exhaling and up with inhaling. Change the position of the legs and then repeat. The duration of this exercise will be five minutes (10 repetitions) (Figure 3). The active arms exercise involves reaching up above the head with one arm that lengthens the muscles from the rib cage. Support it around the elbow with another arm; now the participant needs to activate the arm isometrically for a few seconds. This is to be carried out for a duration of five minutes. This helps in activating the brain for diaphragm relaxation, improving hand-eye coordination and tool-controlling skills (Figure 4). The foot flex exercise and stretch improves posture and socialization. It also helps in relaxation. The participant will be in a sitting position with the ankle of the right leg over the knee of the left leg, placing one hand behind the right knee over the calf and the other hand holding the Achilles tendon. Point and flex the foot for five times for two minutes while holding both hands in the said positions. The energizer helps in improving posture, maintaining back muscle tone and spine suppleness, flexibility, and relaxation. Sit on the chair with a table in front of you, rest your brow on the table while placing your hands on either side of your head, then slowly raise the head until the chin points upwards while inhaling deeply. While exhaling, tuck down and begin to move the head down. Rest the head on the table for a while and breathe deeply. Repeat it five times for the duration of five minutes.

## Results

**Figure 5:**
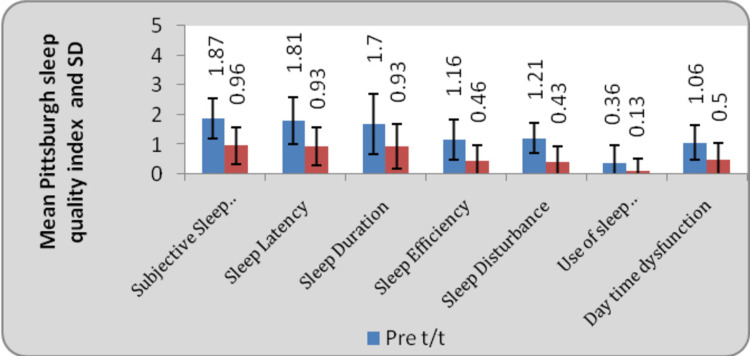
Comparison of Pittsburgh Sleep Quality Index at pre and post-treatment (Wilcoxon signed rank test)

Figure 5 depicts pre and post-treatment scores on the PSQI scale, including all the seven domains which include subjective sleep, sleep latency, sleep duration, sleep efficiency, sleep disturbance, use of sleep medications, and day-time dysfunction. There was an improvement in all of these domains. The most significant improvement was seen in sleep efficiency.

**Figure 6:**
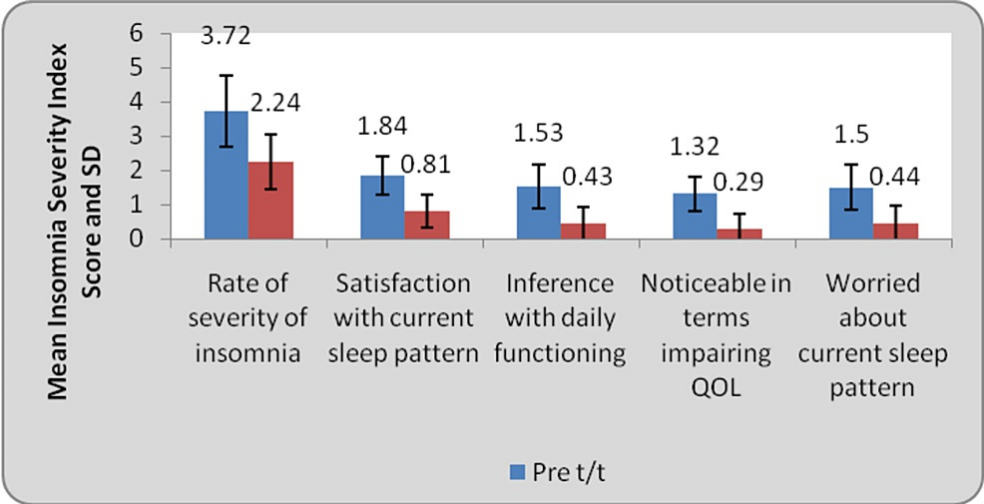
Comparison of Insomnia Severity Index Score at pre and post treatment (Wilcoxo Signed Rank Test)

Figure 6 and Table 2 represent the before and after treatment scores of the Insomnia Severity Index. A total of five domains were assessed for the rate of severity of insomnia, satisfaction with current sleep pattern, interference with daily functioning, noticeable in terms of impairing quality of life, and worry about current sleep pattern. There was improved satisfaction with sleep patterns and improved quality of life.

**Table 2:** Comparison of both outcome measures before and after intervention

Scale	Pittsburgh Sleep Quality Index	Insomnia Severity Index Score	Rho
Pre t/t	9.30±2.11	9.95±1.90	0.709 p=0.0001,S
Post t/t	4.40±1.68	4.21±1.31	0.623 p=0.0001,S

**Figure 7:**
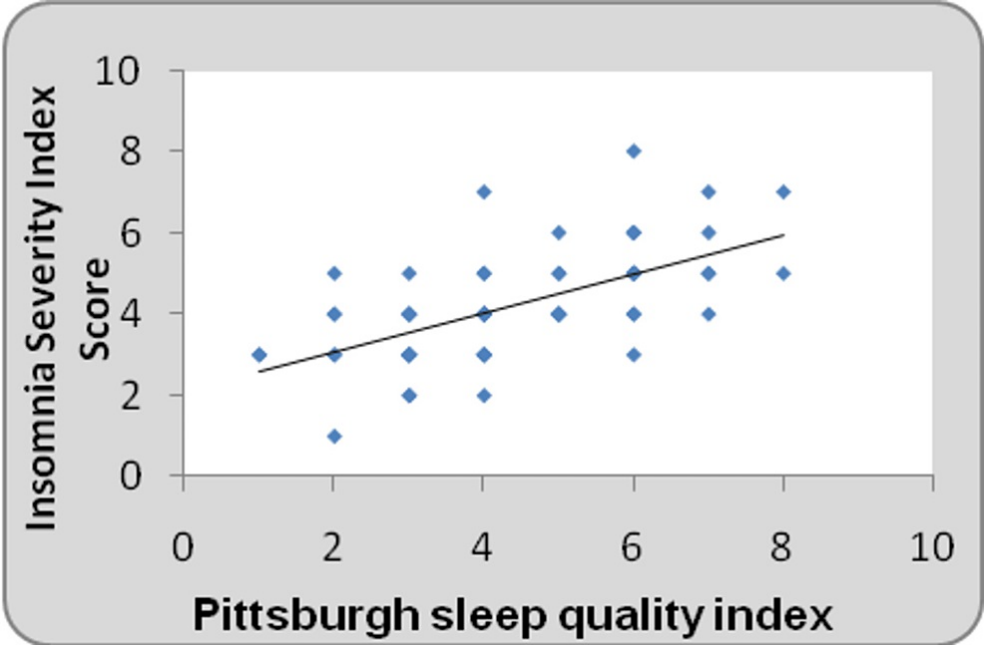
Correlation between Pittsburgh Sleep Quality Index and Insomnia Severity Index Score at post-treatment

Figure 7 represents a comparison of the after-treatment scores of the Insomnia Severity Index and PSQI scales which suggested significant improvement in sleep quality.

The latest version was used to perform statistical analyses. Analysis of variance was used to measure the efficacy of brain gym exercise. Individual studies were checked for homogeneity of the two study groups using the Student's t test. To determine the impact of two steps, all statistical tests were performed with a 95 percent confidence interval (p-value 0.05)

## Discussion

The aim of this study protocol was to assess the effect of brain gym exercises on sleep quality. After having a glance at how to alleviate sleep disturbances in terms of the quality of sleep and being a non-pharmacological method of cure, we found that brain gym exercises can be an answer solvent. Several pieces of research have been done to evaluate the efficacy of brain gym exercises for improving attention, memory, cognition, and inducing relaxation. The outcome was assessed using PSQI and insomnia severity index. The research helped to assess the effectiveness of brain gym exercises on moderate insomniac students and helped with strategies involved to induce relaxation and improve the quality of sleep [8]. In 2019, Effendy et al. studied on effects of brain gym on the quality of sleep and anxiety in the elderly, and found that brain gym significantly improved the quality of sleep and reduced anxiety after eight weeks of intervention. Exercises are proven to act as effective as hypnotic drugs in reducing Insomnia [9].

Preliminary work in this field, most of which was focused on the effectiveness of brain gym as an intervention, was carried out by Spaulding et al. They reported that brain gym exercises have great potential for better learning and scientific understanding [11]. Normal nighttime sleep is divided into two phases, known as non-rapid eye movement sleep and rapid eye movement sleep (REM). Many of the restorative benefits of sleep occur during non-REM sleep. REM sleep, on the other hand, enables the processing of memories and ideas from the day. The phases of sleep advance cyclically from one to four, then to REM, and finally back to stage one. Various neurotransmitters are involved in sleep include dopamine and serotonin. The area of the brain associated with sleep is the hypothalamus, more specifically, the orexin or hypocretin neurons. These neurons directly stimulate the arousal centers as well as the cerebral cortex. Sleep has a restorative effect because it clears the brain from accumulated proteins and possibly neurotoxic waste products that may lead to unhealthy aging via the glymphatic system.

Several approaches for improving sleep quality are used in clinical practice, including relaxation techniques, exercise, acupressure, and medication, but still, brain gym exercises are yet to be included. PSQI is selected as it’s a descriptive scale with a total of seven components that include subjective sleep quality, sleep latency, sleep duration, sleep efficiency, sleep disturbance, use of sleep medications, and day-time changes. It helps to entirely evaluate the patient’s quality of sleep [12].

## Conclusions

The aim of this study was to implement brain gym exercises for improving sleep quality in mild to moderate insomnia, since sleep disturbances are very common among the youth's stressful lives; therefore, a non-pharmacological cure is becoming an increasing necessity. The study suggests that the scope of brain gym exercises must be expanded to other age groups too as results show positive trends. It will not only help in improving sleep quality but improve memory, cognition, and attention and can be used to treat mild to moderate insomnia. The pool of participants can be increased to expand the scope of the research, which will help in establishing a more robust correlation between brain gym and sleep quality. Thus, this study concludes that after the brain gym activity intervention, there was a marked reduction in the score of PSQI. The students involved in the study showed marked improvement in their sleep quality, and it can thus be used as non-pharmacological management for mild to moderate insomnia patients.
